# Predictive value of machine learning for the severity of acute pancreatitis: A systematic review and meta-analysis

**DOI:** 10.1016/j.heliyon.2024.e29603

**Published:** 2024-04-15

**Authors:** Rui Qian, Jiamei Zhuang, Jianjun Xie, Honghui Cheng, Haiya Ou, Xiang Lu, Zichen Ouyang

**Affiliations:** a Department of Gastroenterology, Shenzhen Bao'an Chinese Medicine Hospital, Guangzhou University of Chinese Medicine, Shenzhen 518000, China; b The Fourth Clinical Medical College of Guangzhou University of Chinese Medicine, Shenzhen, 518033, China; c Department of Plumonary and Critical Care Medicine, Shenzhen Bao'an Chinese Medicine Hospital, Guangzhou University of Chinese Medicine, Shenzhen 518000, China; d Department of Hepatology, Shenzhen Bao'an Chinese Medicine Hospital, Guangzhou University of Chinese Medicine, Shenzhen 518000, China

**Keywords:** Machine learning, Acute pancreatitis, Severity, Predictive value, Systematic review

## Abstract

**Background:**

Predicting the severity of acute pancreatitis (AP) early poses a challenge in clinical practice. While there are well-established clinical scoring tools, their actual predictive performance remains uncertain. Various studies have explored the application of machine-learning methods for early AP prediction. However, a more comprehensive evidence-based assessment is needed to determine their predictive accuracy. Hence, this systematic review and meta-analysis aimed to evaluate the predictive accuracy of machine learning in assessing the severity of AP.

**Methods:**

PubMed, EMBASE, Cochrane Library, and Web of Science were systematically searched until December 5, 2023. The risk of bias in eligible studies was assessed using the Prediction Model Risk of Bias Assessment Tool (PROBAST). Subgroup analyses, based on different machine learning types, were performed. Additionally, the predictive accuracy of mainstream scoring tools was summarized.

**Results:**

This systematic review ultimately included 33 original studies. The pooled c-index in both the training and validation sets was 0.87 (95 % CI: 0.84–0.89) and 0.88 (95 % CI: 0.86–0.90), respectively. The sensitivity in the training set was 0.81 (95 % CI: 0.77–0.84), and in the validation set, it was 0.79 (95 % CI: 0.71–0.85). The specificity in the training set was 0.84 (95 % CI: 0.78–0.89), and in the validation set, it was 0.90 (95 % CI: 0.86–0.93). The primary model incorporated was logistic regression; however, its predictive accuracy was found to be inferior to that of neural networks, random forests, and xgboost. The pooled c-index of the APACHE II, BISAP, and Ranson were 0.74 (95 % CI: 0.68–0.80), 0.77 (95 % CI: 0.70–0.85), and 0.74 (95 % CI: 0.68–0.79), respectively.

**Conclusions:**

Machine learning demonstrates excellent accuracy in predicting the severity of AP, providing a reference for updating or developing a straightforward clinical prediction tool.

## 1 Introduction

Acute pancreatitis (AP) ranks among the most common gastrointestinal disorders necessitating acute hospitalization, with a global incidence of approximately 33.74 cases (95 % CI 23.33–48.81) per 100,000 person-years and an estimated mortality rate of about 1.16 (95 % CI 0.85–1.58) per 100,000 person-years [1]. The incidence of AP is on the rise over time, notably in developed regions like Europe and North America [2]. AP is characterized by a local and systemic inflammatory response, and its clinical course varies, with most patients experiencing a self-limiting mild AP that resolves within 1 week [3]. However, approximately 20 % of patients progress to moderate or severe AP, potentially involving pancreas necrosis, peripancreatic tissue necrosis, organ failure, or both, resulting in a mortality rate of 20–40 % [4]. Therefore, predicting the course of AP is significant in clinical practice (see Table 1).

**Table 1:** Basic information on the inclusion of literature.

No	First author	Year of publication	Author's nationality	Study type	Patient source	Total number of cases	Number of SAP cases in training set	Total number of cases in training set	Generation mode of validation set	Number of cases in validation set	Type of model used
1	Xinrui Jin, MB	2021	China	Retrospective	Single-center	300	122	214	Random sampling	86	ANN
2	Hong-Wei Sun	2021	China	Retrospective	Single-center	802	73	234	Random sampling	568	LR
3	Qiao Lin	2019	China	Retrospective	Single-center	259	81	180	Random sampling	79	SVM
4	Hye Won Choi	2018	Korea	Retrospective	Single-center	192	17	115	Random sampling	77	CTA
5	Zhiyong Yang	2015	China	Retrospective	Single-center	603	68	402	Random sampling	201	DT
6	Bodil Andersson	2011	Sweden	Retrospective	Database	340	20	139	Random sampling	201	ANN
7	Wandong Hong	2011	China	Retrospective	Single-center	420	66	280	Random sampling	167	DT,LR
8	Reza Mofidi	2007	UK	Retrospective	Database	664		399	Random sampling	140	ANN
9	Callum B. Pearce	2006	UK	Retrospective	Single-center	265			Random sampling		LR
10	Mary T. Keogan	2002	USA	Retrospective	Single-center	92			Random sampling		ANN
11	X. CAO	2021	China	Prospective	Multicenter	721	33	571	Random sampling	150	LR
12	Shan-Shan He	2022	China	Retrospective	Multicenter	469			Random sampling		LR
13	Wandong Hong	2022	China	Retrospective	Single-center	648	49	487	Random sampling	161	RF,LR
14	Balázs Kui	2022	Hungary	Prospective	Multicenter	4727	70	1184	Random sampling	3543	XGBoost
15	Guang-hua Liu	2022	China	Retrospective	Multicenter	2595	541	2327	External validation	268	LR
16	Rahul Thapa	2021	USA	Retrospective	Single-center	618,494		334,696	Random sampling	37,189	LR, ANN, XGBoost
17	Fei Tian	2022	China	Retrospective	Single-center		92	312	Random sampling		LR
18	Mats L. Wiese	2022	Germany	Retrospective	Multicenter	705			Random sampling		LR
19	Minyue Yin	2022	China	Retrospective	Multicenter	1012	124	796	External validation	212	GBM, XGBoost, RF, GLM, DL, LASSO
20	Rui Zhong	2022	China	Retrospective	Single-center	1860	175	1302	Random sampling	558	LR
21	You Zhou	2022	China	Retrospective	Single-center	441		308	Random sampling	133	LR,RF,SVM, DT, XGBoost
22	Xiao Xu	2020	China	Retrospective	Multicenter	708			External validation	477	LR
23	Wandong Hong	2019	China	Retrospective	Multicenter	894	68	700	External validation	194	LR
24	Jiang-Feng Ye	2017	China	Retrospective	Single-center	302		302			LR
25	Tanka Prasad Bohara	2018	Nepal	Prospective	Single-center	53	7	7			DT
26	Yanmei Zhao	2023	China	Retrospective	Single-center	215	28	141	Random sampling	74	LR
27	Rufa Zhang	2023	China	Retrospective	Multicenter	700	47	499	External validation	201	DL
28	Luo Zhu	2023	China	Retrospective	Multicenter	740	59	631	External validation	109	RF, KNN, DT, NB,AMM
29	Hongyin Liang	2023	China	Retrospective	Single-center	1945	414	1618	Random sampling	180	DL
30	Barrera Gutierrez JC	2023	USA	Prospective	Single-center	516	80	516			DT, LR
31	Bo Li	2023	China	Retrospective	Single-center	436	45	436			LR
32	Deshuai Kong	2023	China	Retrospective	Single-center	212	92	212			LR
33	Zhiyao Chen	2023	China	Retrospective	Single-center	978		783	Random sampling	195	DL

Unfortunately, AP prediction poses a serious challenge. Some scoring tools are available for the early prediction of disease progression in AP, such as clinical and biochemical scoring systems including Acute Physiology and Chronic Health Assessment II (APACHE II) [5], Bedside Index for Severity in Acute Pancreatitis (BISAP) [6], Ranson's Criteria for Pancreatitis Mortality (Ranson's score), and Modified Glasgow Acute Pancreatitis Severity Score (Glasgow's score) [7]. The predictive accuracy of these clinical scoring tools for AP appears to be limited. With the continuous improvement of statistical theory and remarkable advances in computers over the past few years, machine learning has gradually gained popularity and application in clinical practice. Supervised machine learning is frequently used for the diagnosis, prognosis, or prediction of the course of diseases [8,9]. In this context, some investigations have attempted to develop machine learning models to predict the severity of AP.

Nevertheless, the predictive accuracy of different machine-learning models varies. Some models are hardly interpretable but highly accurate, including support vector machines (SVMs), random forests (RF), reinforcement learning (RL), deep learning (DL), and Adaptive Neural Networks (ANN). Conversely, other models are highly interpretable but less accurate, such as decision trees (DT), and logistic regression (LR) [10]. Moreover, the efficiency of predictors plays a crucial role in enhancing the predictive performance of machine-learning models. Currently, there is insufficient systematic evidence to describe the accuracy of machine-learning models in predicting the severity of AP. Hence, this systematic evaluation aims to delve into the accuracy of machine learning in predicting AP severity, providing a reference for updating or developing clinical prediction tools.

## 2 Methods

### 2.1 Study registration

The study followed the Preferred Reporting Items for Systematic Review and Meta-Analysis (PRISMA 2020) guidelines and was registered prospectively in PROSPERO (ID: CRD42023387761).

### 2.2 Eligibility criteria

Inclusion criteria

(1) Participants were diagnosed with AP.
(2) In current research on machine learning to predict disease progression, cohort studies appeared to be more common, although some studies still employed case-control and cross-sectional designs. Consequently, we included cohort studies, case-control studies, and cross-sectional studies in our analysis.
(3) Studies that developed a complete predictive model for the severity of AP were included. Due to the diverse modeling variables in current predictive models (e.g. explainable clinical features, radiomics features, genomics, etc.), our systematic review also included the studies that constructed predictive models with different·modeling variables.
(4) The severity of AP was defined based on Atlanta classification [11,12].
(5) Currently, in machine learning-based predictive models, some original studies did not validate their constructed models using an independent validation set. Studies without independent validation sets were also included in order to analyze whether overfitting existed in the results of machine learning.
(6) For the meta-analysis of machine learning, it was crucial to discuss different modeling variables and predictive performance of various machine learning methods for the development and updating of subsequent scoring tools. Therefore, studies published according to different modeling variables or model types but using the same dataset were also included in the systematic review;
(7) Studies had to be written in English.

Exclusion criteria

(1) Meta-analyses, reviews, guidance, expert opinions, and other similar types of studies were excluded.
(2) Studies conducted only a risk factor or predictors analysis but did not construct a machine-learning model.
(3) Studies did not report any of the following outcome indicators: Roc, c-statistics, c-index, sensitivity, specificity, accuracy, recovery rate, accuracy rate, confusion matrix, diagnostic four-grid table, F1 score.
(4) The stability of predictive models based on few cases was relatively low. Hence, studies with a small sample size (<30 cases) were excluded.
(5) Studies focused solely on the single-factor prediction accuracy were excluded.
(6) Original studies with critically flawed diagnostic criteria for severe pancreatitis, such as those defining the severity of AP based on the length of hospitalization, were excluded.

### 2.3 Source and search strategy

PubMed, EMBASE, Cochrane Library, and Web of Science were systematically searched for original studies on machine learning to predict severe pancreatitis up to November 28, 2022. In order to mitigate the risk of overlooking newly published studies, we conducted a supplementary search in each database on December 5, 2023. The search was conducted using subject terms combined with free words, and there were no restrictions on region or year of publication. The detailed search strategy is illustrated in Table S1.

### 2.4 Study selection and extraction of data

The retrieved studies were imported into Endnote. After removing duplicate studies, we reviewed the titles or abstracts to eliminate irrelevant studies. The full texts of the remaining studies were downloaded and scrutinized to identify eligible studies.

Prior to data extraction, we developed a standardized data extraction spreadsheet. The extracted data encompassed title, first author, publication year, author's country, study type, source of patients, diagnostic criteria for AP, severe pancreatitis case number, total case number, severe pancreatitis case number in the training set, total case number in the training set, validation set generation method, overfitting method, severe pancreatitis case number in the validation set, case number in the validation set, method of dealing with missing values, method of screening/feature selection of the variables, type of used model, as well as modeling variables.

Two investigators (QR, ZJM) independently screened the literature and extracted data, followed by a cross-check. Any disputes were resolved by a third investigator (CHH).

### 2.5 Risk of bias

The risk of bias in the eligible studies was independently assessed by two investigators (QR, ZJM) using PROBAST [8], and their results were cross-checked. Any discrepancies were resolved by a third investigator (CHH).

This tool comprises a set of questions in four different domains: subjects, predictor variables, outcomes, and statistical analyses. The four fields contained two, three, six, and nine unique questions, respectively, answered with Yes/Possibly Yes, No/Probably No, or No Information. If at least one question in a domain was answered with No or Probably No, studies were considered to be at high risk in this domain. If all questions in a domain were answered with Yes/Possibly Yes, studies were considered to have a low risk. If all domains were assessed as having a low risk, the overall risk of bias was considered low; if at least one domain was assessed as having a high risk, the overall risk of bias was high.

### 2.6 Outcomes

The primary outcome indicators included the c-index, which reflected the model's overall accuracy. In addition, sensitivity and specificity were also crucial for assessing model's accuracy. Therefore, the primary outcome indicators also included the sensitivity and specificity of models for predicting severe pancreatitis.

### 2.7 Synthesis methods

A meta-analysis for c-index was conducted. If 95 % confidence intervals (CIs) and standard deviations for the c-index were unavailable in some original studies, we used the equations (Eqs. (1), (2))) provided by Debray TP et al. [13] to estimate their standard deviations. Given the differences in variables and the inconsistent parameters across machine-learning models, the random effects model was prioritized for the meta-analysis of the c-index.

Furthermore, a meta-analysis of sensitivity and specificity was performed using a bivariate mixed-effects model, which required diagnostic fourfold tables. However, this table was not reported in most original studies. Hence, we employed the following two methods to calculate the diagnostic fourfold table: 1. The fourfold table was calculated using precision, sensitivity, specificity, and precision in combination with the number of cases; 2. Sensitivity and specificity were extracted according to the best Youden's index and then the fourfold table· was calculated based on the number of cases. The meta-analysis was conducted using R4.2.0 (R Development Core Team, Vienna, http://www.R-project.org).

(1) $$\text{SE}\left(c\right)\approx \sqrt{\frac{c\left(1-c\right)\left[1+\frac{n^{*}\left(1-c\right)}{2-c}+\frac{m^{*}c}{1+c}\right]}{mn}}$$

(2) $$n^{*}=m^{*}=\frac{m+n}{2}-1$$

Notes: (1) c refers to c-index; (2) n refers to the number of observed events (specifically, the number of severe pancreatitis cases in the training set or validation set); m refers to the total sample size (the number of severe pancreatitis cases in the training set or validation set).

## 3 Results

### 3.1 Study selection

**Fig. 1:**
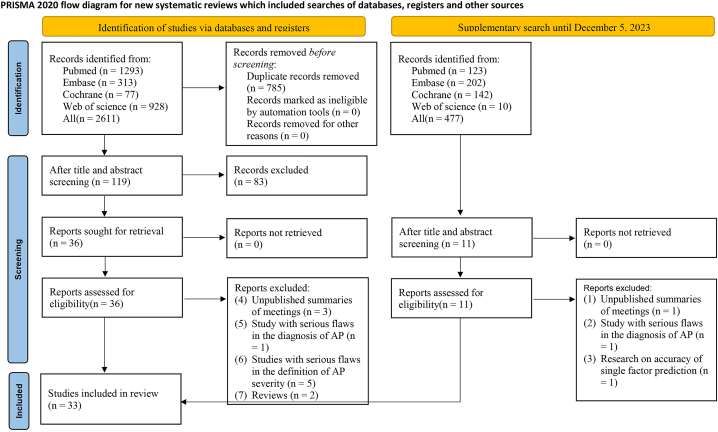
Literature screening process.

We initially retrieved 2611 original studies from the databases. After removing 785 duplicates, 1826 studies were left. Subsequently, after reviewing the titles and abstracts, we identified 36·studies as preliminarily eligible and downloaded their full texts. Then, we excluded three conference abstracts published without peer review, one study with serious flaws in severe pancreatitis·diagnosis, two review articles, and five studies that disagreed on the definition of severity. In addition, the search was updated in 2023, and an additional 477 documents were retrieved. These newly identified studies were screened based on the inclusion and exclusion criteria. Finally, a total of 33 original studies [[14], [15], [16], [17], [18], [19], [20], [21], [22], [23], [24], [25], [26], [27], [28], [29], [30], [31], [32], [33], [34], [35], [36], [37], [38], [39], [40], [41], [42], [43], [44], [45], [46]] were included. The literature screening process is illustrated in Fig. 1.

### 3.2 Study characteristics

The 33 included studies comprised a total of 638,634 AP patients. All eligible studies were cohort studies, only four [23,26,37,43] were prospective cohort studies. These studies were conducted in 8 countries, including 1 [30] in Germany, 1 [26] in Hungary, 1 [17] in South Korea, 1 [37] in Nepal, 1 [18] in Sweden, 2 [20,21] in UK, 3 [22,28,43] in the USA, with the remaining studies conducted in China. Ten [23,24,26,27,30,31,34,35,40,41] studies were multicenter studies, while two studies [18,20] collected subjects from databases. Eleven studies [[14], [15], [16],^18^,[20], [21], [22],^25^,^26^,^31^,^35^] considered overfitting, and k-fold cross-validation was primarily used. The original studies collectively constructed 55 new machine-learning models and evaluated three primary clinical scales: APACHE II, BISAP, and Ranson.

### 3.3 Risk of bias

Regarding the selection of participants, only two studies [18,20] collected participants from registry databases, and four [23,26,37,43] were prospective cohort studies. Others were retrospective cohort studies, which were considered to have a high risk of bias.

In terms of predictors, the predictive factors in all eligible studies appeared to be reasonable; however, three studies [14,18,33] did not characterize the number of missing values, and the interpolated datasets may not be practical when too many values were missing.

In the assessment of outcomes, all included models rationally assessed outcomes. The severity of pancreatitis was defined in a rational manner, and its definition was consistent across the studies. The patient's condition at their admission was used as a modeling variable.

**Fig. 2:**
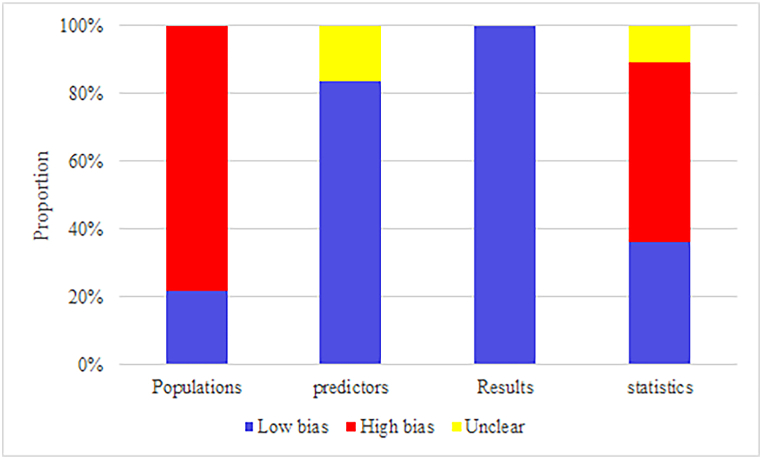
Risk of bias assessment result for included studies.

In terms of statistical methods, ten studies [17,18,25,26,[39], [40], [41],^44^,^45^] had an EPV <10 in the training set, and nine studies [[20], [21], [22],^24^,^30^,^33^,^34^,^36^,^46^] were unable to calculate EPV. In addition, eleven studies [14,16,17,21,22,24,29,30,36,37,39] had no independent validation sets or had independent validation sets with fewer than 100 cases. Two studies [15,18] handled missing values in an unreasonable manner. Five studies [14,[16], [17], [18],^21^,^34^] used a univariate screening method. Eighteen studies [17,19,23,24,[27], [28], [29], [30],[32], [33], [34],[36], [37], [38], [39],[43], [44], [45]] did not consider the overfitting, underfitting, and optimal fitting of the model. The final assessment result is shown in Fig. 2.

### 3.4 Meta-analysis

#### 3.4.1 Newly developed machine-learning models

##### 3.4.1.1 c-Index

**Fig. 3:**
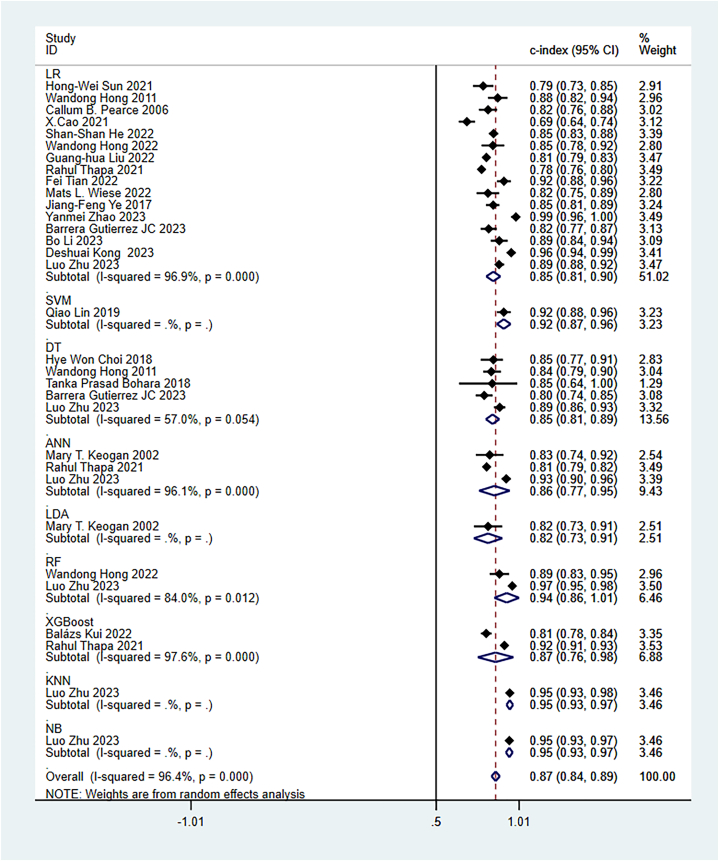
C-index of machine learning model in training set.

**Fig. 4:**
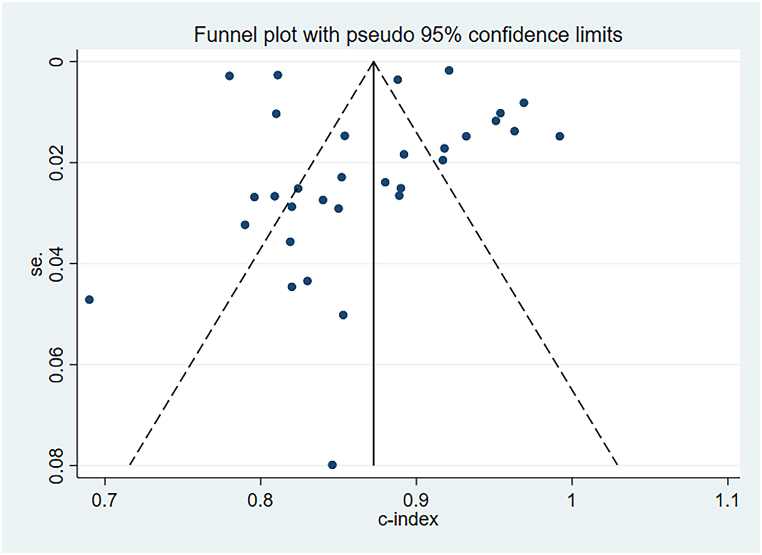
Funnel plot of machine learning model in training set.

The random-effects model was utilized for the meta-analysis of the c-index. The pooled c-index of newly developed models in the training set was 0.87 (95 % CI: 0.84–0.89), and LR was the dominant algorithm with a pooled c-index of 0.85 (95 % CI: 0.81–0.90). RF, SVM, and XGBoost had a better c-index than other models. However, due to the small number of these models, their results needed to be interpreted cautiously (Fig. 3). The funnel plot showed that there was no publication bias in the included studies (Fig. 4).

**Fig. 5:**
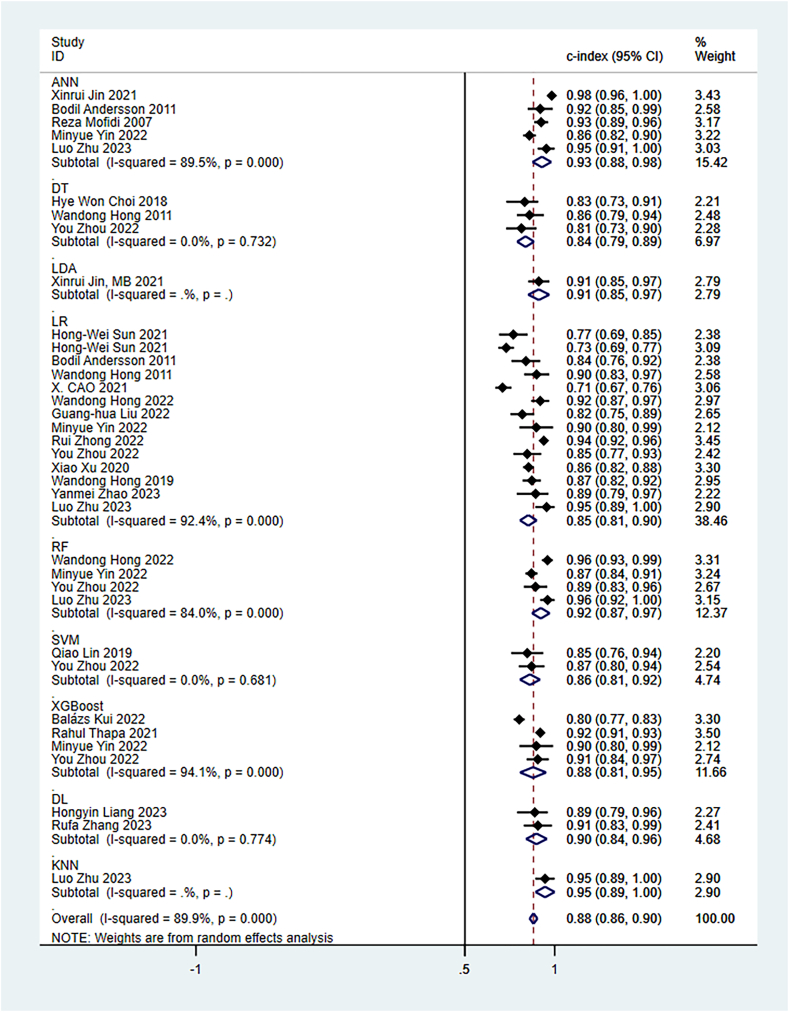
C-index of machine learning model in validation set.

**Fig. 6:**
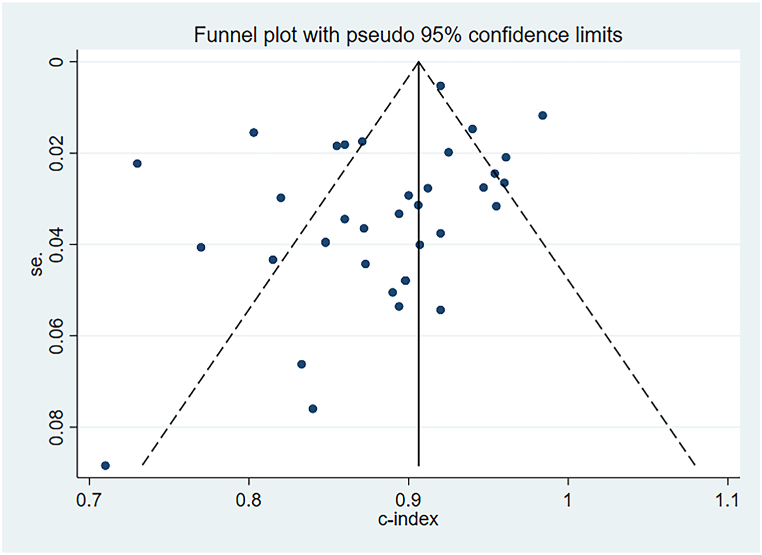
Funnel plot of machine learning model in training set.

In the validation set, the pooled c-index was 0.88 (95 % CI: 0.86–0.90), and LR was the dominant algorithm with a pooled c-index of 0.85 (95 % CI: 0.81–0.92). ANN, Linear Discriminant Approach (LDA), RF, as well as XGBoost had a better c-index than other models. However, due to the limited number of other models, their results needed to be interpreted cautiously (Fig. 5). The funnel plot showed no publication bias in the included studies (Fig. 6).

##### 3.4.1.2 Sensitivity and specificity

**Fig. 7:**
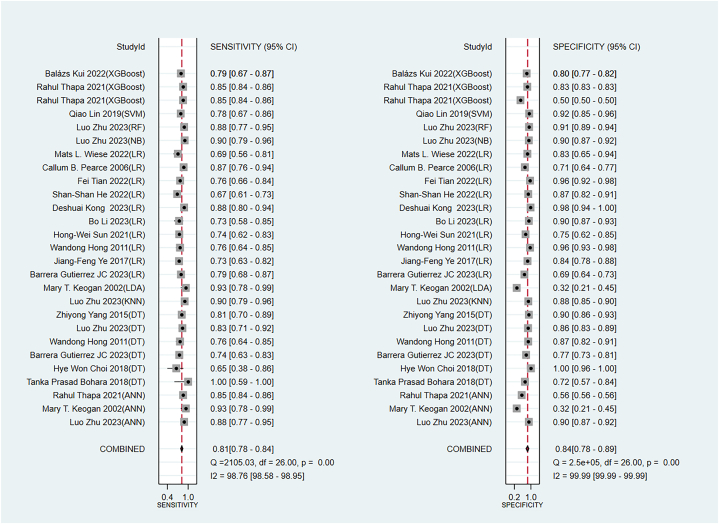
Sensitivity and specificity of machine models in the training set.

**Fig. 8:**
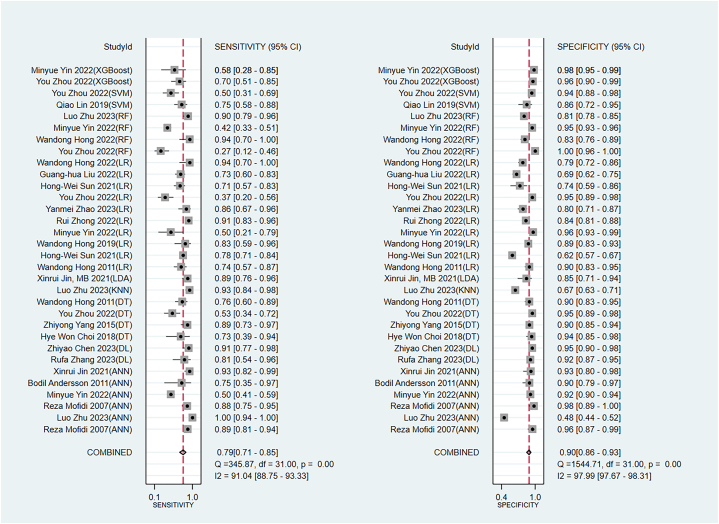
Sensitivity and specificity of machine models in the validation set.

The pooled sensitivity and specificity of the newly developed models in the training set were 0.81 (95 % CI: 0.77–0.84) and 0.84 (95 % CI: 0.78–0.89), respectively (Fig. 7). In the validation set, the pooled sensitivity and specificity were 0.79 (95 % CI: 0.71–0.85) and 0.90 (95 % CI: 0.86–0.93), respectively (Fig. 8).

#### 3.4.2 Established clinical scores

**Fig. 9:**
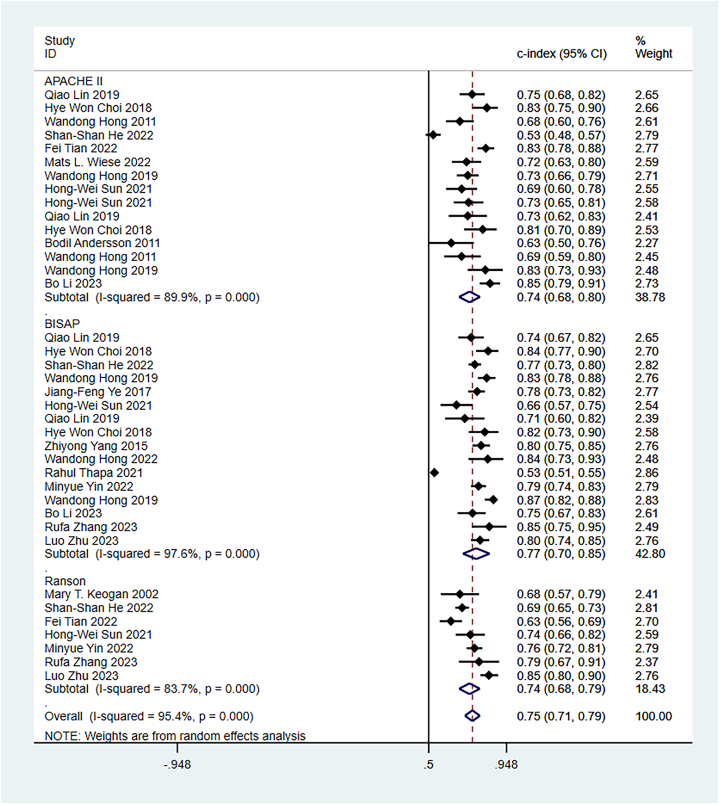
C-index of APACHE II, BIASP and Ranson.

**Fig. 10:**
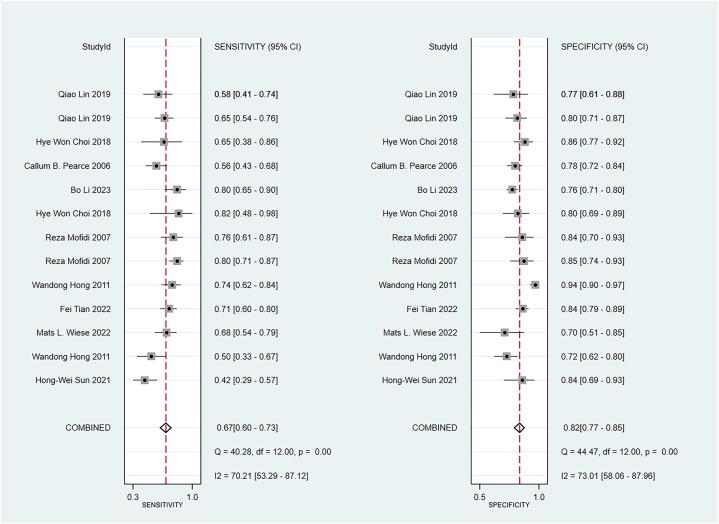
Sensitivity and specificity of APACHE II.

**Fig. 11:**
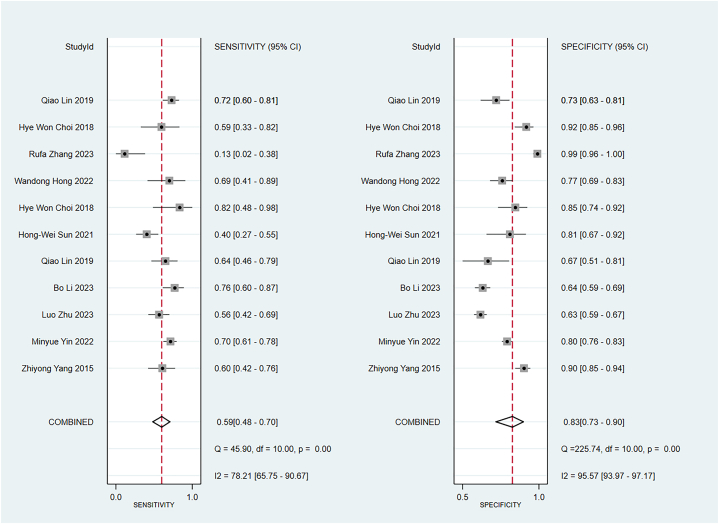
Sensitivity and specificity of BISAP.

**Fig. 12:**
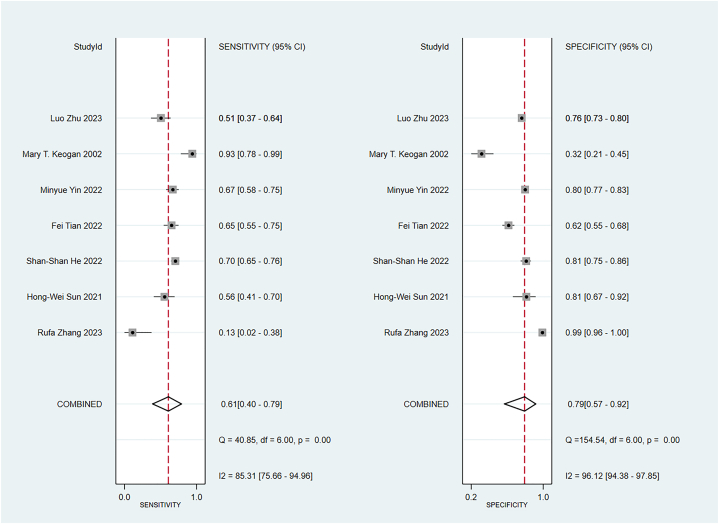
Sensitivity and specificity of Ranson.

Some included studies have verified well-established scoring scales in the training and validation sets. Data were pooled by the random-effects model. The APACHE II showed a c-index of 0.74 (95 % CI: 0.68–0.80), sensitivity of 0.67 (95 % CI: 0.60–0.73), and specificity of 0.82 (95 % CI: 0.77–0.82) (Fig. 9). The BISAP showed a pooled c-index of 0.77 (95 % CI: 0.70–0.85), sensitivity of 0.59 (95 % CI: 0.48–0.70), and specificity of 0.83 (95 % CI: 0.73–0.90). Ranson showed a pooled c-index of 0.74 (95 % CI: 0.68–0.79), sensitivity of 0.61 (95 % CI: 0.40–0.79), and specificity of 0.79 (95 % CI: 0.57–0.92) (Fig. 10, Fig. 11, Fig. 12).

### 3.5 Modeling variables

Modeling variables are crucial for improving the predictive performance of machine-learning models, so we have summarized the modeling variables from the included studies (Table S2).

## 4 Discussion

### 4.1 Summary of the main results/findings

This study demonstrated that the newly developed machine-learning models exhibited desirable accuracy for predicting the severity of AP. Their pooled c-index, sensitivity, and specificity were 0.88 (95 % CI: 0.86–0.90), 0.79 (95 % CI: 0.71–0.85), and 0.90 (95 % CI: 0.86–0.93) in the validation set, respectively. The most commonly used algorithm is LR, which is highly interpretable. However, the LR-based model is less accurate than most of the other models in the validation set.

### 4.2 Comparison with previous reviews

Currently, various assessment methods for predicting the severity of AP are available, including Ranson, BISAP, APACHE-II, CTSI, and CRP24. Previous systematic reviews have explored the predictive accuracy of these assessment tools, but the accuracy remains a matter of debate [[47], [48], [49]]. A recent systematic review reported that the c-index in predicting severity of AP was 0.80 (95 % CI: 0.76–0.85) for CTSI, 0.79 (95 % CI: 0.72–0.86) for BISAP, 0.83 (95 % CI: 0.75–0.91) for mCTSI, 0.73 (95 % CI: 0.64–0.83) for CRP level, 0.81 (95 % CI: 0.75–0.87) for Ranson score, and 0.80 (95 % CI: 0.77–0.83) for APACHE II score [48]. These results suggest that these tools generally exhibit relatively good predictive accuracy. However, it's important to note that the sensitivity of these tools under specific scoring thresholds remains unknown. In our systematic review, the APACHE II score, BISAP, and Ranson score showed a pooled sensitivity of 0.67 (95 % CI: 0.60–0.73), 0.59 (95 % CI: 0.48–0.70), and 0.61 (95 % CI: 0.40–0.79), respectively, indicating that their predictive performance for the severity of AP still needs improvement.

Since AP becomes increasingly prevalent, there is a growing need to focus on predicting its severity. A study by Zhou Y et al. [50] demonstrated that machine-learning methods have the potential to predict severity, complications, death, recurrence, and time to surgery in AP. However, fewer studies specifically focused on severity, and quantitative characterization is lacking. Moreover, some investigations have explored the application of radiomics in the diagnosis and treatment of pancreatitis. A systematic review by Zhou Y et al. [51] indicated that radiomics exhibits desirable accuracy in the differential diagnosis of pancreatitis and pancreatic cancer, although this conclusion is drawn from a limited number of original studies. Another systematic review by Yan et al. [52] suggested that contrast-enhanced MRI may have more favorable accuracy. Tarján D et al. [53] developed an AI-based early prediction tool for the severity of AP; however, this tool has not been validated with a large number of real cases.

In our systematic review, LR was the predominant model utilized algorithm in the included studies. While LR is highly interpretable and can clearly illustrate the relationship between various factors and outcome events, its predictive efficiency remains uncertain. For the early prediction of AP severity, ANN, LDA, RF, SVM, and XGBoost demonstrated superior c-indexes; specifically, ANN, RF, SVM, and XGBoost exhibited highly desirable predictive performance. However, these models were less interpretable. Therefore, selecting an appropriate model presented a challenge for clinicians. Subsequent studies should aim to balance both interpretability and predictive performance.

In the meta-analysis of machine learning, the impact of heterogeneity on results is unavoidable. As previously discussed, the varying accuracy of different machine learning models in detecting outcome events contributes significantly to heterogeneity. In order to explore the source of heterogeneity, subgroup analysis was conducted based on different types of models. Moreover, within the same model, model's structure should be considered, such as the number of hidden layers and neurons in artificial neural networks, the number of decision trees in random forests, and the type of kernel function in support vector machines. However, it is noteworthy that many original studies do not provide detailed descriptions of these aspects, posing challenges for interpreting the meta-analysis results. Additionally, predictive factors may introduce partial heterogeneity, and our study summarizes the included predictive factors. Importantly, our analysis revealed no significant publication bias in the c-index of the training set and validation set, enhancing the reliability of our results.

### 4.3 Advantages and limitations

This study systematically evaluates the predictive accuracy of machine learning models for the severity of AP. However, there are certain limitations. Firstly, the predominant inclusion of retrospective cohort studies might introduce inherent biases. Secondly, although a considerable number of original studies employed independent validation sets to validate the models, a limited proportion of studies conducted multicenter external validation, potentially affecting the generalizability of the findings. Thirdly, some original studies had a small number of cases available for model training, particularly falling short of the recommended EPV>20. Fourthly, the number of included studies remains limited, and certain types of models are only reported in a small number of studies. To conduct a sensitivity analysis, the modeling method should be consistent. Therefore, we were unable to conduct a sensitivity analysis. Fifthly, we include only four prospective cohort studies, which use different modeling methods. Due to the small number of studies, we were unable to perform subgroup analysis based on study type to evaluate the impact of different study types on the results.

## 5 Conclusions

Machine learning demonstrated a relatively satisfactory accuracy in predicting the severity of AP, with certain less interpretable machine-learning models showing particularly promising results. While existing tools have some predictive values, their performance could be enhanced by using large sample sizes and machine learning.

## Ethics approval and consent to participate

Not applicable.

## Funding

The authors declare that they did not receive any funding from any source.

## Consent for publication

Not applicable.

## Data availability

Data included in article/supp. material/referenced in article.

## CRediT authorship contribution statement

**Rui Qian:** Writing – review & editing, Writing – original draft, Visualization, Validation, Supervision, Software, Resources. **Jiamei Zhuang:** Writing – review & editing, Writing – original draft, Funding acquisition, Formal analysis, Data curation, Conceptualization. **Jianjun Xie:** Writing – review & editing, Writing – original draft, Resources, Project administration, Methodology, Investigation. **Honghui Cheng:** Writing – review & editing, Writing – original draft, Visualization, Validation, Supervision, Software. **Haiya Ou:** Writing – review & editing, Writing – original draft, Resources, Funding acquisition, Conceptualization. **Xiang Lu:** Writing – review & editing, Writing – original draft, Validation, Project administration, Data curation. **Zichen Ouyang:** Writing – review & editing, Writing – original draft, Supervision, Investigation, Data curation.

## Declaration of competing interest

The authors declare that they have no competing interests.

## References

[1] Xiao A.Y. (2016). Global incidence and mortality of pancreatic diseases: a systematic review, meta-analysis, and meta-regression of population-based cohort studies. Lancet Gastroenterol. Hepatol..

[2] Iannuzzi J.P. (2022). Global incidence of acute pancreatitis is increasing over time: a systematic review and meta-analysis. Gastroenterology.

[3] Johnson C.D., Besselink M.G., Carter R. (2014). Acute pancreatitis. BMJ.

[4] Boxhoorn L. (2020). Acute pancreatitis. Lancet.

[5] Larvin M., McMahon M.J. (1989). Apache-II score for assessment and monitoring of acute pancreatitis. Lancet.

[6] Wu B.U. (2008). The early prediction of mortality in acute pancreatitis: a large population-based study. Gut.

[7] Mounzer R. (2012). Comparison of existing clinical scoring systems to predict persistent organ failure in patients with acute pancreatitis. Gastroenterology.

[8] Andaur Navarro C.L. (2021). Risk of bias in studies on prediction models developed using supervised machine learning techniques: systematic review. BMJ.

[9] Fleuren L.M. (2020). Machine learning for the prediction of sepsis: a systematic review and meta-analysis of diagnostic test accuracy. Intensive Care Med..

[10] Gunning D. (2019). XAI-Explainable artificial intelligence. Sci. Robot..

[11] Banks P.A. (2013). Classification of acute pancreatitis--2012: revision of the Atlanta classification and definitions by international consensus. Gut.

[12] Bradley E.L. (1993). A clinically based classification system for acute pancreatitis. Summary of the International Symposium on Acute Pancreatitis, Atlanta, Ga, September 11 through 13, 1992. Arch. Surg..

[13] Debray T.P. (2019). A framework for meta-analysis of prediction model studies with binary and time-to-event outcomes. Stat. Methods Med. Res..

[14] Jin X. (2021). Comparison of MPL-ANN and PLS-DA models for predicting the severity of patients with acute pancreatitis: an exploratory study. Am. J. Emerg. Med..

[15] Sun H.W. (2021). Accurate prediction of acute pancreatitis severity with integrative blood molecular measurements. Aging.

[16] Lin Q. (2020). Radiomics model of contrast-enhanced MRI for early prediction of acute pancreatitis severity. J. Magn. Reson. Imag..

[17] Choi H.W. (2018). Early prediction of the severity of acute pancreatitis using radiologic and clinical scoring systems with classification tree analysis. AJR Am. J. Roentgenol..

[18] Andersson B. (2011). Prediction of severe acute pancreatitis at admission to hospital using artificial neural networks. Pancreatology.

[19] Hong W. (2011). Prediction of severe acute pancreatitis using classification and regression tree analysis. Dig. Dis. Sci..

[20] Mofidi R. (2007). Identification of severe acute pancreatitis using an artificial neural network. Surgery.

[21] Pearce C.B. (2006). Machine learning can improve prediction of severity in acute pancreatitis using admission values of Apache II score and C-reactive protein. Pancreatology.

[22] Keogan M.T. (2002). Outcome analysis of patients with acute pancreatitis by using an artificial neural network. Acad. Radiol..

[23] Cao X. (2021). Establishment and verification of a nomogram for predicting severe acute pancreatitis. Eur. Rev. Med. Pharmacol. Sci..

[24] He S.S. (2022). Establishment of early multi-indicator prediction models of moderately severe acute pancreatitis and severe acute pancreatitis. Gastroenterol. Res. Pract..

[25] Hong W. (2022). Usefulness of random forest algorithm in predicting severe acute pancreatitis. Front. Cell. Infect. Microbiol..

[26] Kui B. (2022). EASY-APP: an artificial intelligence model and application for early and easy prediction of severity in acute pancreatitis. Clin. Transl. Med..

[27] Liu G.H. (2022). Development and validation of a nomogram for early assessment the severity of acute pancreatitis. Scand. J. Gastroenterol..

[28] Thapa R. (2022). Early prediction of severe acute pancreatitis using machine learning. Pancreatology.

[29] Tian F. (2022). Correlation between severity of illness and levels of free triiodothyronine, interleukin-6, and interleukin-10 in patients with acute pancreatitis. Med. Sci. Mon. Int. Med. J. Exp. Clin. Res..

[30] Wiese M.L. (2022). Identification of early predictors for infected necrosis in acute pancreatitis. BMC Gastroenterol..

[31] Yin M. (2022). Automated machine learning for the early prediction of the severity of acute pancreatitis in hospitals. Front. Cell. Infect. Microbiol..

[32] Zhong R. (2022). Development and evaluation of a nomogram to predict the eventual severity of the first episode of acute pancreatitis. Pancreas.

[33] Zhou Y. (2022). Prediction of the severity of acute pancreatitis using machine learning models. Postgrad. Med..

[34] Xu X., Ai F., Huang M. (2020). Deceased serum bilirubin and albumin levels in the assessment of severity and mortality in patients with acute pancreatitis. Int. J. Med. Sci..

[35] Hong W. (2019). Development and validation of a risk prediction score for severe acute pancreatitis. J. Transl. Med..

[36] Ye J.F. (2017). Building and verifying a severity prediction model of acute pancreatitis (AP) based on BISAP, MEWS and routine test indexes. Clin. Res. Hepatol. Gastroenterol..

[37] Bohara T.P. (2018). Prospective validation of a decision tree model for prediction of severity in acute pancreatitis. J. Nepal Health Res. Counc..

[38] Yang Z. (2015). Prediction of severe acute pancreatitis using a decision tree model based on the revised Atlanta classification of acute pancreatitis. PLoS One.

[39] Zhao Y. (2023). Early prediction of acute pancreatitis severity based on changes in pancreatic and peripancreatic computed tomography radiomics nomogram. Quant. Imag. Med. Surg..

[40] Zhang R. (2023). Application value of the automated machine learning model based on modified computed tomography severity index combined with serological indicators in the early prediction of severe acute pancreatitis. J. Clin. Gastroenterol..

[41] Luo Z. (2023). Development and evaluation of machine learning models and nomogram for the prediction of severe acute pancreatitis. J. Gastroenterol. Hepatol..

[42] Liang H. (2023). Predicting acute pancreatitis severity with enhanced computed tomography scans using convolutional neural networks. Sci. Rep..

[43] Barrera Gutierrez J.C. (2023). Severe acute pancreatitis prediction: a model derived from a prospective registry cohort. Cureus.

[44] Li B. (2023). Establishment and validation of a nomogram prediction model for the severe acute pancreatitis. J. Inflamm. Res..

[45] Kong D. (2023). A novel HCP (heparin-binding protein-C reactive protein-procalcitonin) inflammatory composite model can predict severe acute pancreatitis. Sci. Rep..

[46] Chen Z. (2023). Deep learning models for severity prediction of acute pancreatitis in the early phase from abdominal nonenhanced computed tomography images. Pancreas.

[47] Gao W., Yang H.X., Ma C.E. (2015). The value of BISAP score for predicting mortality and severity in acute pancreatitis: a systematic review and meta-analysis. PLoS One.

[48] Mikó A. (2019). Computed tomography severity index vs. Other indices in the prediction of severity and mortality in acute pancreatitis: a predictive accuracy meta-analysis. Front. Physiol..

[49] Yang Y.X., Li L. (2016). Evaluating the ability of the Bedside index for severity of acute pancreatitis score to predict severe acute pancreatitis: a meta-analysis. Med. Princ. Pract..

[50] Zhou Y. (2022). Machine learning predictive models for acute pancreatitis: a systematic review. Int. J. Med. Inf..

[51] Zhong J. (2022). A systematic review of radiomics in pancreatitis: applying the evidence level rating tool for promoting clinical transferability. Insights Imaging.

[52] Yan G. (2022). Radiomics and its applications and progress in pancreatitis: a current state of the art review. Front. Med..

[53] Tarján D., Hegyi P. (2022). Acute pancreatitis severity prediction: it is time to use artificial intelligence. J. Clin. Med..

