# Supplementary material for: Predictive value of machine learning for the severity of acute pancreatitis: A systematic review and meta-analysis
Source: Heliyon. 2024 Apr 15;10(8):e29603. doi: 10.1016/j.heliyon.2024.e29603 (PMC11035062; doi:10.1016/j.heliyon.2024.e29603)
Supplement: Multimedia component 1 [file mmc1.docx]

**Table S1** Literature search strategy

**1.Pubmed**

| Search number | Query | Results |
| --- | --- | --- |
| #1 | ((((((((((((Pancreatitis[Title/Abstract]) OR (Acute Edematous Pancreatitides[Title/Abstract])) OR (Acute Edematous Pancreatitis[Title/Abstract])) OR (Pancreatic Parenchymal Edema[Title/Abstract])) OR (Pancreatic Parenchymal Edemas[Title/Abstract])) OR (Pancreatic Parenchyma with Edema[Title/Abstract])) OR (Acute Pancreatitis[Title/Abstract])) OR (Acute Pancreatitides[Title/Abstract])) OR (Peripancreatic Fat Necrosis[Title/Abstract])) OR (Peripancreatic Fat Necroses[Title/Abstract])) OR (hereditary pancreatitis[Title/Abstract])) OR (pancreatic inflammation[Title/Abstract])) OR (traumatic pancreatitis[Title/Abstract]) | 65,572 |
| #2 | "Machine Learning"[Mesh] | 51,474 |
| #3 | ((((((((((((((((((((((machine learning[Title/Abstract]) OR (Transfer Learning[Title/Abstract])) OR (Deep learning[Title/Abstract])) OR (Ensemble Learning[Title/Abstract])) OR (artificial intelligence[Title/Abstract])) OR (Prediction model[Title/Abstract])) OR (random forest[Title/Abstract])) OR (neural network[Title/Abstract])) OR (neural networks[Title/Abstract])) OR (CNN[Title/Abstract])) OR (ANN[Title/Abstract])) OR (Support vector machine[Title/Abstract])) OR (SVM[Title/Abstract])) OR (Gradient Boosting Machine[Title/Abstract])) OR (GBM[Title/Abstract])) OR (Nomogram[Title/Abstract])) OR (XGBoost[Title/Abstract])) OR (Decision tree[Title/Abstract])) OR (ResNet-50[Title/Abstract])) OR (ResNet[Title/Abstract])) OR (Naive Bayesian[Title/Abstract])) OR (Risk Prediction[Title/Abstract])) OR (Risk-Prediction[Title/Abstract]) | 273,395 |
| #4 | "Pancreatitis"[Mesh] | 55,617 |
| #5 | ("Pancreatitis"[Mesh]) OR (((((((((((((Pancreatitis[Title/Abstract]) OR (Acute Edematous Pancreatitides[Title/Abstract])) OR (Acute Edematous Pancreatitis[Title/Abstract])) OR (Pancreatic Parenchymal Edema[Title/Abstract])) OR (Pancreatic Parenchymal Edemas[Title/Abstract])) OR (Pancreatic Parenchyma with Edema[Title/Abstract])) OR (Acute Pancreatitis[Title/Abstract])) OR (Acute Pancreatitides[Title/Abstract])) OR (Peripancreatic Fat Necrosis[Title/Abstract])) OR (Peripancreatic Fat Necroses[Title/Abstract])) OR (hereditary pancreatitis[Title/Abstract])) OR (pancreatic inflammation[Title/Abstract])) OR (traumatic pancreatitis[Title/Abstract])) | 75,117 |
| #6 | ("Machine Learning"[Mesh]) OR (((((((((((((((((((((((machine learning[Title/Abstract]) OR (Transfer Learning[Title/Abstract])) OR (Deep learning[Title/Abstract])) OR (Ensemble Learning[Title/Abstract])) OR (artificial intelligence[Title/Abstract])) OR (Prediction model[Title/Abstract])) OR (random forest[Title/Abstract])) OR (neural network[Title/Abstract])) OR (neural networks[Title/Abstract])) OR (CNN[Title/Abstract])) OR (ANN[Title/Abstract])) OR (Support vector machine[Title/Abstract])) OR (SVM[Title/Abstract])) OR (Gradient Boosting Machine[Title/Abstract])) OR (GBM[Title/Abstract])) OR (Nomogram[Title/Abstract])) OR (XGBoost[Title/Abstract])) OR (Decision tree[Title/Abstract])) OR (ResNet-50[Title/Abstract])) OR (ResNet[Title/Abstract])) OR (Naive Bayesian[Title/Abstract])) OR (Risk Prediction[Title/Abstract])) OR (Risk-Prediction[Title/Abstract])) | 278,411 |
| #7 | (("Machine Learning"[Mesh]) OR (((((((((((((((((((((((machine learning[Title/Abstract]) OR (Transfer Learning[Title/Abstract])) OR (Deep learning[Title/Abstract])) OR (Ensemble Learning[Title/Abstract])) OR (artificial intelligence[Title/Abstract])) OR (Prediction model[Title/Abstract])) OR (random forest[Title/Abstract])) OR (neural network[Title/Abstract])) OR (neural networks[Title/Abstract])) OR (CNN[Title/Abstract])) OR (ANN[Title/Abstract])) OR (Support vector machine[Title/Abstract])) OR (SVM[Title/Abstract])) OR (Gradient Boosting Machine[Title/Abstract])) OR (GBM[Title/Abstract])) OR (Nomogram[Title/Abstract])) OR (XGBoost[Title/Abstract])) OR (Decision tree[Title/Abstract])) OR (ResNet-50[Title/Abstract])) OR (ResNet[Title/Abstract])) OR (Naive Bayesian[Title/Abstract])) OR (Risk Prediction[Title/Abstract])) OR (Risk-Prediction[Title/Abstract]))) AND (("Pancreatitis"[Mesh]) OR (((((((((((((Pancreatitis[Title/Abstract]) OR (Acute Edematous Pancreatitides[Title/Abstract])) OR (Acute Edematous Pancreatitis[Title/Abstract])) OR (Pancreatic Parenchymal Edema[Title/Abstract])) OR (Pancreatic Parenchymal Edemas[Title/Abstract])) OR (Pancreatic Parenchyma with Edema[Title/Abstract])) OR (Acute Pancreatitis[Title/Abstract])) OR (Acute Pancreatitides[Title/Abstract])) OR (Peripancreatic Fat Necrosis[Title/Abstract])) OR (Peripancreatic Fat Necroses[Title/Abstract])) OR (hereditary pancreatitis[Title/Abstract])) OR (pancreatic inflammation[Title/Abstract])) OR (traumatic pancreatitis[Title/Abstract]))) | 313 |

**2.Embase**

| Search number | Query | Results |
| --- | --- | --- |
| #1 | 'pancreatitis'/exp | 121256 |
| #2 | pancreatitis:ab,ti OR 'acute edematous pancreatitides':ab,ti OR 'acute edematous pancreatitis':ab,ti OR 'pancreatic parenchymal edema':ab,ti OR 'pancreatic parenchymal edemas':ab,ti OR 'pancreatic parenchyma with edema':ab,ti OR 'acute pancreatitis':ab,ti OR 'acute pancreatitides':ab,ti OR 'peripancreatic fat necrosis':ab,ti OR 'peripancreatic fat necroses':ab,ti OR 'hereditary pancreatitis':ab,ti OR 'pancreas inflammation':ab,ti OR 'pancreatic inflammation':ab,ti OR 'traumatic pancreatitis':ab,ti | 98062 |
| #3 | #1 OR #2 | 131257 |
| #4 | 'machine learning'/exp | 348948 |
| #5 | 'machine learning':ab,ti OR 'transfer learning':ab,ti OR 'deep learning':ab,ti OR 'ensemble learning':ab,ti OR 'artificial intelligence':ab,ti OR 'prediction model':ab,ti OR 'random forest':ab,ti OR 'neural network':ab,ti OR 'neural networks':ab,ti OR cnn:ab,ti OR ann:ab,ti OR 'support vector machine':ab,ti OR svm:ab,ti OR 'gradient boosting machine':ab,ti OR gbm:ab,ti OR nomogram:ab,ti OR xgboost:ab,ti OR 'decision tree':ab,ti OR 'resnet 50':ab,ti OR resnet:ab,ti OR 'naive bayesian':ab,ti OR 'risk聽prediction':ab,ti OR 'risk prediction':ab,ti | 444041 |
| #6 | #4 OR #5 | 629495 |
| #7 | #3 AND #6 | 1293 |

**3.Web of science**

| Search number | Query | Results |
| --- | --- | --- |
| #1 | Pancreatitis (Topic) OR Acute Edematous Pancreatitides (Topic) OR Acute Edematous Pancreatitis (Topic) OR Pancreatic Parenchymal Edema (Topic) OR Pancreatic Parenchymal Edemas (Topic) OR Pancreatic Parenchyma with Edema (Topic) OR Acute Pancreatitis (Topic) OR Acute Pancreatitides (Topic) OR Peripancreatic Fat Necrosis (Topic) OR Peripancreatic Fat Necroses (Topic) OR hereditary pancreatitis (Topic) OR pancreas inflammation (Topic) OR pancreatic inflammation (Topic) OR traumatic pancreatitis (Topic) | 74844 |
| #2 | machine learning (Topic) OR Transfer Learning (Topic) OR Deep learning (Topic) OR Ensemble Learning (Topic) OR Prediction model (Topic) OR random forest (Topic) OR neural network (Topic) OR neural networks (Topic) OR CNN (Topic) OR ANN (Topic) OR Support vector machine (Topic) OR SVM (Topic) OR Gradient Boosting Machine (Topic) OR GBM (Topic) OR Nomogram (Topic) OR XGBoost (Topic) OR Decision tree (Topic) OR ResNet-50 (Topic) OR ResNet (Topic) OR Naive Bayesian (Topic) OR Risk Prediction (Topic) OR Risk-Prediction (Topic) | 1979521 |
| #3 | #2 AND #1 | 928 |

**Table S2**

| **No.** | **First author** | **Year** | **Modeling variables** |
| --- | --- | --- | --- |
| 1 | Xinrui Jin, MB | 2021 | CRP;LYM-R;NEU;NEU-R;NLR;PAMY;WBC;AMY |
| 2 | Hong-Wei Sun | 2021 | Cr;PT;TG;K^+^;ALT;BUN;TT;PCT;ALB;A/G;LY% |
| 3 | Qiao Lin | 2019 | Contrast(*θ*=135°，d=1); Correlation(*θ*=45°，d=7); Compactness1; ConvexHullVolume; ConvexHullVolume3D; Mass; Mean Breadth; Number Of Voxel; SurfaceArea; Volume; ShortRunHighGrayLevelEmpha(*θ*=0) |
| 4 | Hye Won Choi | 2018 | APACHE II score; BISAP score; Balthazar grade; EPIC score |
| 5 | Zhiyong Yang | 2015 | Cr;LDH;OI |
| 6 | Bodil Andersson | 2011 | duration of pain until arrival at the emergency department;CR;Hb;ALT;RR;WBC |
| 7 | Wandong Hong | 2011 | pleural effusion;Ca^2+^;BUN |
| 8 | Reza Mofidi | 2007 | Age; Hypotension unresponsive to fluid resuscitation; SIRS; SaO2; LDH; Glu; Ure; Ca2+; HCT; WBC |
| 9 | Callum B. Pearce | 2006 | Age, CRP, RR, PaO_2_；pH；Cr; WBC;GCS score |
| 10 | Mary T. Keogan | 2002 | BP, extent of inflammation, fluid aspiration,Cr, Ca^2+^, and the presence of concurrent severe illness |
| 11 | X. CAO | 2021 | Sex, Ca^2+^, Cr, Neut%, Lymph% and Eo% |
| 12 | Shan-Shan He | 2022 | WBC; LDH; CRP; TG; D-dimer; K+; Ca^2+^ |
| 13 | Wandong Hong | 2022 | BUN; Cr; Alb; HDL-C; LDL-C; Ca^2+;^ Glu |
| 14 | Balázs Kui | 2022 | RR; T; abdominal muscular reflex; gender; age; Glu |
| 15 | Guang-hua Liu | 2022 | Alb; Ca^2+^; NLR; SIRS |
| 16 | Rahul Thapa | 2021 | SpO2; BMI; LP; WBC; HCT; diagnosis of abdominal pain at admission time; |
| 17 | Fei Tian | 2022 | FT3; IL-6; IL-10 |
| 18 | Mats L. Wiese | 2022 | Cr; CRP; Alb; alcoholic etiology |
| 19 | Minyue Yin | 2022 | NEU; Cr; LDH; TGs; INR; RCR; CAR; SIRS; PE |
| 20 | Rui Zhong | 2022 | RDW; D-dimer; APOA1; Alb |
| 21 | You Zhou | 2022 | CTSI score; ALB; LDH; NEUT |
| 22 | Xiao Xu | 2020 | SOFA score; HGB; Alb; TBIL; UN |
| 23 | Wandong Hong | 2019 | SIRS; Alb; BUN; PE |
| 24 | Jiang-Feng Ye | 2017 | BISAP; Ca^2+^ |
| 25 | Tanka Prasad Bohara | 2018 | Cr; LDH; OI |
| 26 | Yanmei Zhao | 2023 | Radiomics |
| 27 | Rufa Zhang | 2023 | WBC, Ca, MCTSI (Modified Computed Tomography Severity Index) |
| 28 | Luo Zhu | 2023 | CRP, WBC, NLR, ALT, AST, ALB, GLU, BUN, CR, CA, HDL, PI, PN, extrapancreatic complication |
| 29 | Hongyin Liang | 2023 | Radiomics |
| 30 | Barrera Gutierrez JC | 2023 | Cr, WBC, PCT, and SIRS |
| 31 | Bo Li | 2023 | Neutrophil, albumin, blood glucose, serum calcium, serum creatinine, blood urea nitrogen, and procalcitonin |
| 32 | Deshuai Kong | 2023 | BMI, Leukocytes, INR, HBP, CRP, PCT |
| 33 | Zhiyao Chen | 2023 | Radiomics |
